# Extraction process optimization of polyphenols from Indian *Citrus sinensis –* as novel antiglycative agents in the management of diabetes mellitus

**DOI:** 10.1186/2251-6581-13-11

**Published:** 2014-01-07

**Authors:** Asaithambi Shakthi Deve, Thiyagarajan Sathish kumar, Kuppamuthu Kumaresan, Vinohar Stephen Rapheal

**Affiliations:** 1 Department of Biotechnology, Kumaraguru College of Technology, Coimbatore 641 049, Tamil Nadu, India

**Keywords:** *Artocarpus heterophyllus*, *Citrus sinensis*, Diabetes mellitus, Response surface methodology, Quercetin

## Abstract

**Background:**

Diabetes mellitus is a chronic metabolic disorder characterized by increased blood glucose level. It has become an epidemic disease in the 21st century where, India leads the world with largest number of diabetic subjects. Non-enzymatic glycosylation (glycation) is severe form of diabetes, occurs between reducing sugar and proteins which results in the formation of advanced glycation end products (AGEs) that leads to the other complicated secondary disorders. In this context, *Mangifera indica* (Mango), *Syzygium cumini* (Jambul), *Vitis vinifera* (Grapes), *Citrus sinensis* (Orange), *Artocarpus heterophyllus* (Jackfruit), *Manilkara zapota* (Sapodilla) seeds were evaluated for their antiglyation activity. Attempts were made to isolate the polyphenols in the seeds that have recorded the maximum activity.

**Methods:**

Different extraction methods (shake flask, centrifugation and pressurized hot water) using various extractants (organic solvents, hot water and pressurized hot water) were adopted to investigate the *in vitro* antiglycation activity. Central composite (CCD) design based Response Surface Methodology (RSM) was espoused to optimize the extraction process of polyphenols from the fruit seeds that have recorded poor antiglycation activity. The PTLC analysis was performed to isolate the polyphenols (Flavonoids and phenolic acids) and LC-PDA-MS analysis was done for structure prediction.

**Results:**

Pressurized hot water extraction of *Artocarpus heterophyllus* (87.52%) and *Citrus sinensis* seeds (74.79%) was found to possess high and low antiglycation activity, respectively. The RSM mediated optimization process adopted for the *Citrus sinensis* seeds have revealed that 1:15 solvent ratio (hexane to heptane), 6 minutes and 1:20 solid to liquid ratio as the optimal conditions for the extraction of polyphenols with a maximum antiglycation activity (89.79%). The LC-PDA-MS analysis of preparative thin layer chromatography (PTLC) eluates of *Artocarpus heterophyllus* seed has showed the presence of compounds like quercetin (301.2), 4-hydroxy phenyl acetic acid (149.0), rhamnosyl-di-hexosyl quercetin sulphate (857.6), quercetin-3-O-xyloside (428.2), rutin (613.4), diosmetin (298.1) and luteolin (283.0).

**Conclusion:**

The *Artocarpus heterophyllus* was observed to possess a significant antiglycation activity and the activity of *Citrus sinensis* was improved after the optimization process, which proved that both the seeds may be used as a traditional medicine in the management of chronic diabetes mellitus.

## Introduction

Diabetes mellitus, a metabolic endocrine disorder, has become a common global health problem that affects >170 million people worldwide. It is one of the leading causes of death and disability. It is estimated that by 2030, the number will rise to 366 million. The majority of diabetes (~90%) is type 2 diabetes (T2D) caused by a combination of impaired insulin secretion from pancreatic beta cells and insulin resistance of the peripheral target tissues, especially muscle and liver [1]. Uncontrolled diabetes can complicate pregnancy and birth defects. Generally, the long-term complications of diabetes are categorized into macrovascular (coronary artery disease, peripheral arterial disease, and stroke) and microvascular (diabetic nephropathy, neuropathy, and retinopathy) [2].

High glucose level in the blood can be subjected for a spontaneous reaction that occurs between the aldehyde group and amino groups of the protein chain that leads to the formation of the Schiff base. This intermediate subsequently undergo a number of biochemical reactions to yield various fluorescent brown pigments known as advanced glycation end products [3]. The oxidation process is believed to play an important role in formation of AGEs. Further oxidation of AGEs leads to the formation of intermediate carbonyl compounds that can react with the nearby lysine or arginine residues to form protein crosslinks and therefore, agents with antioxidant property may retard the process of AGEs formation and their subsequent oxidation progression. Recently, role of fruits in the prevention of glycation activity has gained a substantial attention among scientific community and various families like Anarcardiaceae, Curcubitaceae, Moraceae, Annonaceae, Sapotaceae, Phyllanthus, Myrtaceae, Rutaceae etc. have been documented for their noteworthy role to own antioxidant and antiglycation activities. Several natural compounds like curcumin, rutin and garcinol have been reported to possess antioxidant property as well their role in the prevention of formation of AGEs *in vitro* and *in vivo*[4].

The investigation of antidiabetic agents of plant origin which are used in traditional medicine is of great importance. The seed kernel of *Mangifera indica* is one such herbal source which is mentioned in Ayurvedic literature for treating Diabetes mellitus. The kernel is astringent, anti-helmintic, stimulant, anti-inflammatory, antibacterial, antifungal, anti-pasmodic, anti-scorbutic and is administered in asthma, diabetes, nasal bleeding, diarrhea and ulcers [5]. The bark, leaves and seed extracts of *Syzygium cumini* have been reported to possess anti-inflammatory, anti-bacterial and anti-diarrheal effect [6]. *Vitis vinifera* seed have a variety of health benefits like antibacterial, antiviral, anticarcinogenic, anti-inflammatory, antioxidant and vasodilatory actions [7]. Similarly, *Artocarpus heterophyllus* possess numerous medicinal properties such as antibacterial, antioxidant, antidiabetic, anti-inflammatory, anti-diuretic, immunomodulatory and have been useful in the treatment of fever, skin diseases, convulsions, constipation, ophthalmic disorders and snake bite. The seeds were added to flour and baked or boiled/roasted and consumed as nutritious diet [8]. *Citrus sinensis*, belongs to Rutaceae family possess antioxidant, antibacterial, antifungal, anti-carcinogenic, anti-ulcer, anti-anxiety, antidiabetic and anti-inflammatory properties [9]. *Manilkara zapota* is a plant with traditional and medical importance. It contains several compounds like phenolics, alkaloids, epicatechin, leucocyanidin, leucodelphinidin, leucopelargonidin, chlorogenic acid, gallic acid, sugars, carotenoids and minerals like calcium, iron, zinc, copper and potassium. It has documented several properties like antibacterial, antifungal, antipyretic, antioxidant, antiulcer and used in the treatment of fever, wounds, diarrohea and pulmonary ailments [10].

Even though the antidiabetic activity has been reported in the plant species, no work on antiglycation has been explored in the above mentioned plants. In this milieu, our laboratory have focused on the evaluation of *in vitro* antiglycation activity in the seeds of *Mangifera indica*, *Syzygium cumini*, *Vitis vinifera*, *Citrus sinensis*, *Artocarpus heterophyllus* and *Manilkara zapota*.

## Materials and methods

### Chemicals

Bovine serum albumin (Fraction V), Glucose, Sodium azide and Nitro-blue tetrazolium was obtained from Himedia bioscience company Ltd., India.

### Seed collection

*Mangifera indica, Artocarpus heterophyllus, Manilkara zapota, Citrus sinensis, Syzygium cumini* and *Vitis vinifera* fruits were obtained from the local market. The seeds were removed, dried at 60˚C for 5days, fine powdered and used for the experimental analysis.

### Preparation of hot water extract (HWE)

In a clean dry 250 ml conical flask, 0.5 g of powdered material was weighed and extracted with 25 ml of distilled water by placing it in a boiling water bath at temperature 90˚C for 5 minutes. The content was filtered using Whatman No.2 filter paper, the filtrate obtained is precipitated by adding 10% of ammonium sulphate and centrifuged at 5000 rpm for 10 minutes. The supernatant were used to investigate the *in vitro* antiglycation activity.

### Preparation of organic solvent extract

In a clean dry 250 ml of separating funnel, 5 g of powdered material was added to 20 ml of petroleum ether and shook thoroughly for 15 minutes. The resultant mixture was filtered and the filtrate was used for the antiglycation assay. The obtained residue was then re-extracted with chloroform, acetone, ethyl acetate, ethanol and distilled water, respectively and the process was repeated as mentioned above for each solvent extract. Each of the content was filtered using Whatman No.2 filter paper, the filtrate obtained is precipitated by adding 10% of ammonium sulphate and centrifuged at 5000 rpm for 10 minutes. The supernatant used for the experimental analysis [11].

### Preparation of pressurized hot water extract (PHWE)

About 0.5 g of powdered material was weighed in a clean dry pressurized vessel and added 5 ml of distilled water. The vessel is placed in an oil bath at 180˚C, 1002 kPa for 10 minutes. The resultant mixture was filtered using Whatman No.2 filter paper, the filtrate obtained is precipitated by adding 10% of ammonium sulphate and centrifuged at 5000 rpm for 10 minutes and the supernatant was used for the assay.

### *In vitro* antiglycation assay

A slightly modified method proposed by [12] was adopted for the evaluation of antiglycation activity. About 10 ml of BSA (20 mg/ml), 5 ml of glucose (500 mM) and 0.02% of sodium azide, dissolved in phosphate buffer (200 mM, pH 7.4) was added to 5 ml of the test sample and incubated at 37 C for 5 days. A mixture of BSA, glucose and sodium azide was used as a control along with the test sample. After 5days, 0.5 ml of the glycated material was mixed with 2 ml of nitro-blue tetrazolium (0.3 mM) dissolved in sodium carbonate buffer (100 mM, pH 10.35), incubated at room temperature for 15minutes and the absorbance was read spectrophotometrically (Beckman DU-530) at 530 nm.

### Estimation of total phenolic content (TPC) by Folin-Ciocalteau method

A method proposed by [13] was adopted to determine the total phenolic content. To 0.1 ml of the extract, added 3.9 ml of distilled water and 0.5 ml of Folin’s reagent. The tube was incubated at room temperature for 3 minute. To this added 2 ml of 20% sodium carbonate and kept at boiling water bath for 1 minute. The blue color formed was read at 650 nm. Gallic acid was used as a standard for constructing a calibration curve.

### Two-dimensional thin layer chromatography (TLC) and preparative thin layer chromatography (PTLC)

The glass plates (20 × 10 cm) were coated with the silica gel (0.1-0.2 mm thickness) with the help of an applicator and dried for 24 hrs to remove moisture or water and other adsorbed substances from the surface, in order to activate the plate. 40 μl of test sample was spotted from 2 cm above the base of the plate and a two dimensional thin layer chromatogram was developed using ethyl acetate: formic acid: acetic acid: distilled water (100: 11: 11: 26) and 15% acetic acid as mobile phase solvents. The plates were dried before spraying liquid ammonia (spraying agent) and visualized under long UV light at 360 nm to detect the presence of flavonoids [14]. The PTLC analysis was performed according to the procedure described by Meena and Patni, [15].

### LC-PDA-MS (ESI+) analysis

The liquid chromatography electron spray mass spectrometry (LC-MS) analysis was performed on Varian Inc, (USA) 410 Prostar Binary LC with 500 MS IT PDA Detectors. The column was C18, 250 × 4.6 mm, i.d. 5 μm. The mobile phase A was made up of acetonitrile, while B was made of 0.1% formic acid (pH 4.0, adjusted with ammonium hydroxide). The gradient elution was performed at 1 ml/min with an initial condition of 12% of mobile phase A and 88% of mobile phase B for 10 min. The mobile phase A was linearly increased from 15% to 100% and analysis was performed from 20 minutes to 95 minutes. The eluates were monitored by PDA (Multi wavelength) detector at 260 nm. About 20 μl of the PTLC *Artocarpus heterophyllus* seed eluate was introduced into the ESI source and the mass spectra were scanned in the range 100-1000 amu and the maximum ion injection time was set at 200nS. Ion spray voltage was set at 5.3 KV and capillary voltage 34 V. The MS scan ran up to 26.67 minutes.

### Optimization of polyphenols using response surface methodology (RSM)

The optimum extraction condition for the polyphenols (flavonoids and phenolic acids) was determined by central composite design (CCD) based response surface methodology that includes three variables and three factorial levels (For a total of 20 runs, 4 runs were dummy variables). The experimental design is a full factorial CCD with 8 factorial cube points, 6 axial points and 6 centre points in cube (Table 1). The independent variables used in this study were hexane: heptane ratio (v/v), extraction time (minutes) and solid: liquid ratio (g/ml). A single factor analysis of variance was adopted to investigate the effect of each factor on the extraction of polyphenols. Second-order polynomial equation was used to express the investigated responses (Y) as a function of the coded independent variables, where X1, X2…X3 are the independent variables affecting the responses Y, β0, βj, (i = 1, 2…k), βii (i = 1, 2…k), and βij (i = 1, 2…k; j = 1, 2…k) are regression coefficients for intercept, linear, quadratic, and interaction terms, respectively; k is the number of variables. Coded independent variables for our experiment are solvent ratio, time and solid: liquid ratio.

(1) $$\begin{array}{l} Y=\beta 0+{\Sigma^{K}}_{i=1}\beta i\;\mathrm{Xi}+{\Sigma^{K}}_{i=1}\beta {{\mathrm{ii}\;X}_{i}}^{2}\\ \;+{\Sigma^{K}}_{i=1}{;}_{i<j}{\Sigma^{K}}_{j=2}\beta_{\mathrm{ij}}X_{i}X_{j} \end{array}$$

**Table 1:** Real values adopted in full factorial central composite design (CCD)

**Std order**	**Hexane: heptane**	**Time (min)**	**Solid: liquid**
1	1:5	2	1:10
2	1:15	2	1:10
3	1:5	6	1:10
4	1:15	6	1:10
5	1:5	2	1:20
6	1:15	2	1:20
7	1:5	6	1:20
8	1:15	6	1:20
9	1:6	4	1:15
10	1:18	4	1:15
11	1:10	0.6	1:15
12	1:10	7	1:15
13	1:10	4	1:6.6
14	1:10	4	1:23.4
15	1:10	4	1:15
16	1:10	4	1:15
17	1:10	4	1:15
18	1:10	4	1:15
19	1:10	4	1:15
20	1:10	4	1:15

### Statistical analysis

Statistical analysis was performed using Minitab 15 (trial version). The results were statistically tested by the analysis of variance (ANOVA) at 5% level of significance. Response surface and contour plots were generated and analyzed.

## Results and discussion

The experimental analysis on the evaluation of the antiglycation property has revealed that *Artocarpus heterophyllus* (PHWE: 87.52%) and *Mangifera indica* (PHWE: 84.76%) possess maximal activity, *Vitis vinifera* (solvent extraction: 83.31%) and *Manilakara zapota* (PHWE: 81.59%) own near maximal activity and *Syzygium cumini* (solvent extraction: 78.14%) and *Citrus sinensis* (solvent extraction: 75.65%) hold near moderate or considered as poor activity. On the comparison of different extraction methodologies like HWE, PHWE and solvent extraction, it was observed and recorded that HWE (41.65 ± 13.95%) was inferior, and both solvent based (77.7 ± 6.78%) and PHWE (76.70 ± 20.98%) was superior in the extraction of phytochemicals that were responsible for the antiglycation activity. A Comparison of *in vitro* antiglycation activity of six different seeds using different extraction methods has been depicted in Table 2. One way ANOVA analysis has revealed that there is a significant difference at 5% level (p < 0.05), between the seeds in the antiglycation activity (Table 3).

**Table 2:** **Comparison of *in vitro *** **antiglycation of the seeds using different extraction methods**

**S. No.**	**Name of the seed**	**Hot water extraction (%)**	**Pressurized hot water extraction (%)**	**Solvent extraction (%)**
1.	*Syzygium cumini*	16.07	49.24	78.14
2.	*Citrus sinensis*	19.80	74.79	75.65
3.	*Mangifera indica*	59.54	84.76	65.17
4.	*Vitis vinifera*	68.87	79.86	83.31
5.	*Manilakara zapota*	44.72	81.59	81.24
6.	*Artocarpus heterophyllus*	40.92	**87.52**	82.68

**Table 3:** One way ANOVA analysis of the antiglycation activity between different seeds

**ANOVA table**	**Sum of squares**	**Degrees of freedom**	**Mean square**	**F-value**
Treatment (between columns)	8331	5	1666	3.562
Residual (within columns)	16842	36	467.8	
Total	25173	41	2133.8	

Medicinal plants, now-a-days, gain significant attention among research community due of its greater exploitation by the common public for the better prevention and cure of various ailments. Because of the adverse and or toxic effects of synthetic drugs sizeable awareness has been created on par with natural remedies which are safe, effective and non-toxic. The traditional techniques of solvent extraction of plant based materials are typically based on the extraction type, choice of right solvents, temperature, extraction type, agitation and material ratio for improved extraction efficiency as well as the mass transfer [16].

*Artocarpus heterophyllus* is generally recommended by traditional medical practitioners as a classical treatment for diabetes mellitus. Significant reduction of blood glucose (49%) through the administration of flavonoid fraction of the leaf in the alloxan induced diabetic rats has been reported. Moreover, no significant necrosis of hepatic, renal and cardiac tissues was observed and documented which, proved the non-toxicity of the leaves [17]. Similarly, investigation conducted by [18] has documented the efficacy of the hypoglycemic effect of *Artocarpus heterophyllus* leaves and the studies have proved a noteworthy reduction in the blood glucose and total cholesterol. Studies conducted by [19] have revealed the potent antidiabetic activity of *Artocarpus heterophyllus* fruits and its remarkable impact in the increase of glucose tolerance. Furthermore, investigation in the leaves of *Artocarpus heterophyllus* have classically indicated its efficiency in the attenuation of glycosylation of hemoglobin, enhancement in the transport of glucose across cells, stimulation of insulin release and inhibition of cholesterol biosynthetic enzymes [20,21]. The present results on the significant and superior effect of the antiglycation property of PHWE of *Artocarpus heterophyllus* seeds was well corroborated with the above mentioned scientific documentation.

RSM is defined as a statistical technique adopted for any quantitative data from suitable experimental designs to determine and solve the multivariate equations [22]. Application of RSM in the optimization process may save cost, time and energy. Central composite design (CCD) is widely used for fitting a second order method in response surface method which consisted of four runs at the corners of the square, four runs at the center of this square and four axial runs [23]. It used to estimate parameters of a full second order polynomial model. CCD had been introduced by Box and William on 1957 which was divided into three point group of design; factorial, axial point and centre point.

**Figure 1:**
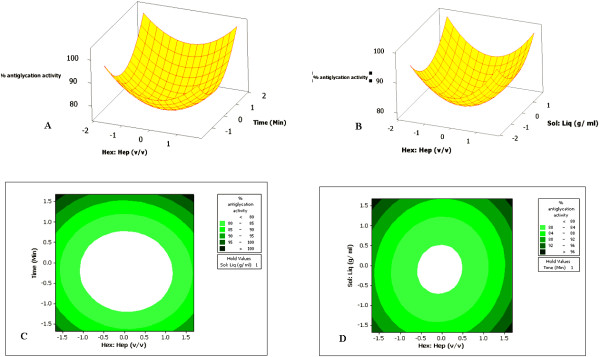
**Response surface and contour diagrams of antiglycation activity (%) of *Citrus sinensis *** **seeds as a function of: (A, C) Hexane: heptane and time, (B, D) Solid: liquid and Hexane: heptane.**

**Figure 2:**
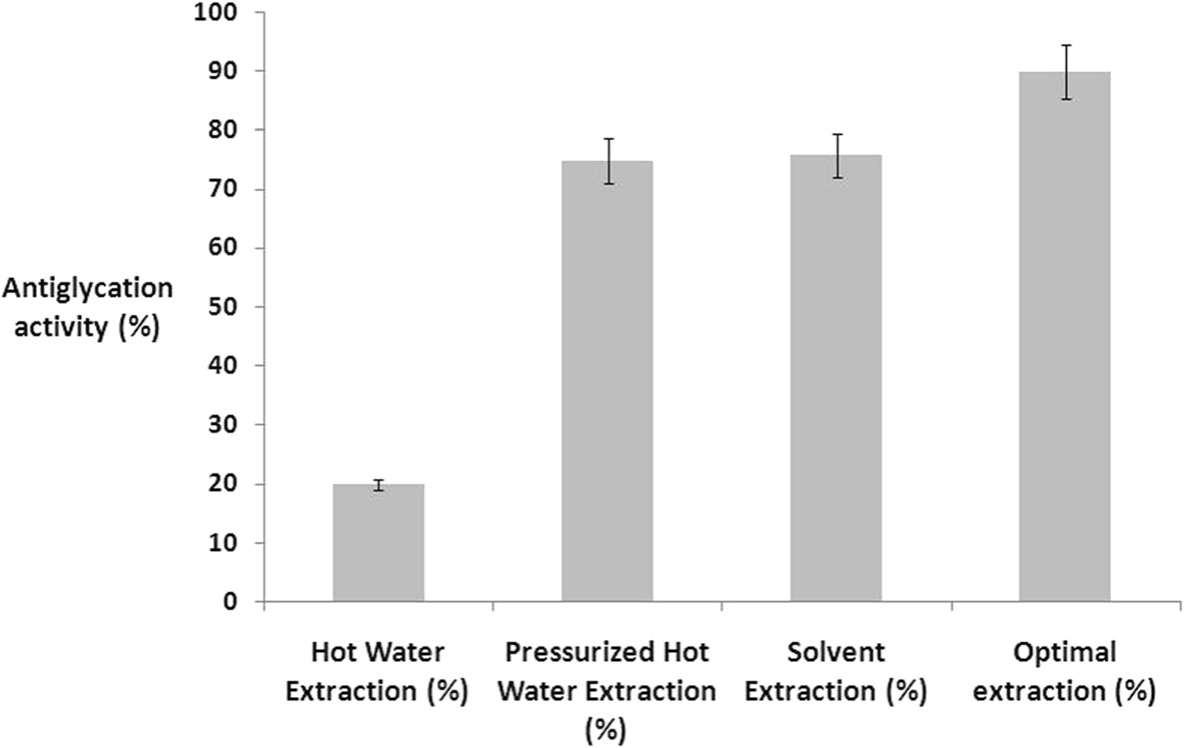
**Comparison of antiglycation activity of *Citrus sinensis *** **seeds by different extraction methods.**

In the present study, a suitable central composite based experimental design (CCD) has been formulated in order to develop an empirical model to investigate the interactions of the selected variables responsible for the extraction of polyphenols (flavonoids and phenolic acids) from the seeds of *Citrus sinensis*. The surface and contour diagrams depicted in Figure 1 have indicated that maximum antiglycation activity (89.79%) was obtained under the optimal conditions (extraction of polyphenols) at 1: 15 (hexane: heptane ratio, (v/v)), 6 minutes (Time), and 1: 20 (solid: liquid ratio (g/ml)) (Table 4). A significant increase (15%) in the antiglycation activity of the optimal seed extract of *Citrus sinensis* has been observed compared with hot water, pressurized hot water and solvent extracts (Figure 2). From multiple regression analysis, it was observed that the second-order polynomial equation generated from the experimental design can explain the antiglycation activity (%) regardless of the significance of coefficients:

$$\begin{array}{l} Y=79.7176+3.2012x_{1}+3.1435x_{2}\\ \;-2.2808x_{3}-0.8322{x_{1}}^{2}+0.4353{x_{2}}^{2}+0.9957{x_{3}}^{2}\\ \;-3.6975x_{1}x_{2}+1.4225x_{1}x_{3}+4.1952x_{2}x_{3} \end{array}$$

where, Y represents the dependent variable (% antiglycation activity) and *x*_*1*_, *x*_*2*_ and *x*_*3*_ represents the independent ratio, time and solid–liquid ratio). The goodness of fit manifested by determination coefficient (R^2^ = 0.90) showed that the sample variation of 90% for antiglycation activity is characteristic to the independent variables and only 10% of the total variation cannot be explained by the developed model. The positive coefficient of hexane-heptane ratio (*x*_*1*_) and time (*x*_*2*_) revealed a linear effect in the increased antiglycation activity, whereas, the negative coefficient of solid: liquid ratio (*x*_*3*_) indicated an inverse effect (i.e., increase in the ratio leads to decreased antiglycation activity and vice versa). The cross-product results clearly showed that the most significant interaction has occurred between hexane-heptane ratio (*x*_*1*_) and time (*x*_*2*_), and also equally between time (*x*_*2*_) and solid: liquid ratio (*x*_*3*_). All the three variables mentioned above were found to significant at 5% level (p < 0.05) (Table 5).

**Table 4:** Central composite design (CCD) experimental results of real values

**Std order**	**Hexane: heptane**	**Time (min)**	**Solid: liquid**	**% antiglycation**	**Total phenolic content (mg/g tissue)**
1	1:5	2	1:10	85.38	0.70
2	1:15	2	1:10	86.89	0.70
3	1:5	6	1:10	80.00	0.68
4	1:15	6	1:10	87.24	0.72
5	1:5	2	1:20	84.27	0.69
6	1:15	2	1:20	88.97	0.72
7	1:5	6	1:20	88.76	0.72
8	1:15	6	1:20	89.79	0.72
9	1:6	4	1:15	85.31	0.69
10	1:18	4	1:15	77.66	0.63
11	1:10	0.6	1:15	81.72	0.68
12	1:10	7	1:15	87.72	0.72
13	1:10	4	1:6.6	85.86	0.71
14	1:10	4	1:23.4	72.34	0.61
15	1:10	4	1:15	78.69	0.64
16	1:10	4	1:15	72.84	0.61
17	1:10	4	1:15	74.34	0.63
18	1:10	4	1:15	71.38	0.61
19	1:10	4	1:15	69.17	0.59
20	1:10	4	1:15	74.21	0.61

**Table 5:** One way ANOVA analysis of the variables: (a) hexane: heptane ratio, (b) time and (c) solid: liquid ratio

**Source**	**DF**	**SS**	**MS**	**F**	**P**
**Hex: hep (v/v)**	4	464.1	116.0	4.25	0.017
**Error**	15	410.0	27.3		
**Total**	19	874.1			
**(a)**					
**Time (min)**	4	512.0	128.0	5.30	0.007
**Error**	15	362.1	24.1		
**Total**	19	874.1			
**(b)**					
**Sol: liq (g/ml)**	4	488.1	122.0	4.74	0.011
**Error**	15	386.0	25.7		
**Total**	19	874.1			

Apparently, it has been proved that the experimental runs at centre points (run No.10 and 14–20) didn’t record the maximum antiglycation activity and this may be because of the inverse effect of solid: liquid ratio. The recovery yield of polyphenols through its solubility in the system can be highly influenced by the solvent system adopted. Solvent polarity plays a pivotal role in the increasing solubility of polyphenols. As per the previous reports by [24-27], mostly aqueous mixtures of ethanol, methanol, acetonitrile and acetone at various volume ratios were significant in the extraction of polyphenols. However, contradictory results in the selection of solvent, i.e., high non-polar solvents like hexane have been also explored and documented its efficiency in the extraction process [28]. A similar conflicting result was observed and recorded in our present investigation.

In order to understand the extraction kinetics of polyphenols effectively, it is an appropriate choice in the experimental design to incorporate the time variability parameter. This may avoid an increase loss of polyphenols, provides maximal antiglycation activity and also to find out the time range that leads to a minimal variability in the recovery of polyphenols [29]. Generally, longer the extraction time may leads to the loss of the polyphenols via oxidation. The oxidized products may polymerize to form insoluble compounds and inhibit the diffusion of the polyphenols. According to Fick’s law, this could be because of the existence of equilibrium between the solute and the solvent [30]. Hence, prolonged extraction time is futile, grab a lot of energy, cost and in our case, the extraction time was found to be very low (6 minutes). Previous reports [31,32] on the recovery of flavonoids from the Citrus peels have revealed a maximum of 30 minutes and 3 hrs optimal extraction time, respectively and our present investigation in this dimension was well correlated with the above mentioned scientific findings.

The effect of solid/liquid ratio has a positive outcome in our present study, i.e., enhanced antiglycation activity due to increased extraction of polyphenols at higher ratio (1: 20). Mass transfer is the principle phenomenon, where a concentration gradient may be larger at high solid/liquid ratio, which greatly influences the dissolution of the solutes from the sample matrix to the solvent [33].

**Figure 3:**
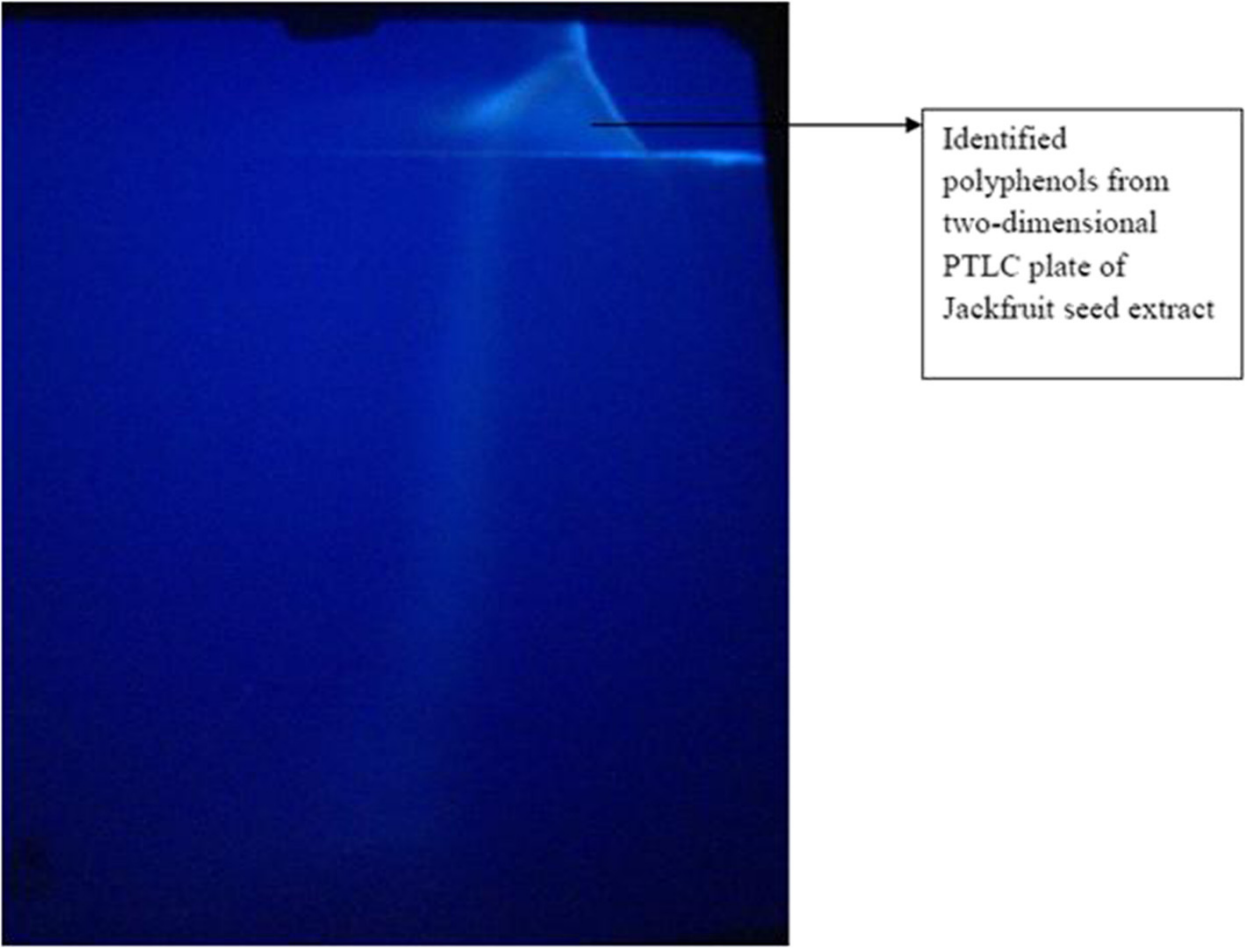
**Isolation of polyphenols (flavonoids and phenolic acids) from PHWE of *Artocarpus heterophyllus *** **by 2D PTLC and its visualization under far UV light.**

TLC is used for the analysis of several phytochemicals. The advantages of using TLC to construct the fingerprints of herbal medicines are its simplicity, versatility, high velocity, specific sensitivity and simple sample preparation. Thus, TLC is a convenient method of determining the quality and possible adulteration of herbal products [34]. Both the seed extracts viz., *Artocarpus heterophyllus* and *Citrus sinensis*, respectively has revealed the presence of polyphenols that includes flavonols, flavonoids glycosides and phenolic acids. Due to the maximal antiglycation activity, *Artocarpus heterophyllus* seeds were subjected for PTLC and further mass spectrometry analysis in the prediction of various polyphenolic compounds. The identification of flavonoids and phenolic acids in the 2D PTLC eluate of *Artocarpus heterophyllus* seeds was depicted in Figure 3 and the antiglycation activity was found to be 95.38%, which is about 8% increase in the activity compared to PHWE *Artocarpus heterophyllus.* This substantiates a strong role of polyphenols like flavonoids in the attenuation of glycation activity.

LC-PDA-MS (ESI+) Analysis of *Artocarpus heterophyllus* PTLC eluate have revealed the presence of compounds like rutin, quercetin, quercetin-3-O-xyloside, 4-hydroxy phenyl acetic acid, rhamnosyl-di-hexosyl quercein sulphate, luteolin and diosmetin (Table 6).

**Table 6:** **Retention time (R**_**t**_**), molecular ions [M + H]**^**+**^**, fragment ions, molecular weight and identified compounds in the PTLC eluate (flavonols, flavonoids glycosides and phenolic acids scanned at 260 nm (PDA))**

**S. No.**	**Retention time (R**_**t, **_**min)**	**MS *m/z *** **[M + H]**^**+**^	***m/z *** **fragment ions**	**Molecular weight**	**Identified compound**
1.	2.5	149	-	151	4-hydroxy phenyl acetic acid
2.	5.0	283	-	286.2	Luteolin
3.	6.0	298.1	-	299	Diosmetin
4.	6.5	301.2	149/121/93	301.2	Quercetin
5.	9.5	428.2	149/301.2	434.8	Quercetin-3-O-xyloside
6.	14	613.4	301.2/149/121/93	610.5	Rutin
7.	20.5	857.6	-	853	Rhamnosyl-di-hexosyl quercein sulphate

*Artocarpus* is an important genus in the Family of Moraceae and the phytochemical constituents distributed in this genus are mainly flavonoids, especially flavones, isoflavones and chalcones, xanthones and prenylated stilbenes. So far, seventy prenylated flavonoids have been isolated and possess a wide range of biological properties [35]. Previously, Omar et al., [36] has isolated isosquercitrin from the leaves of *Artocarpus heterophyllus* and explained its potent antioxidant, antidiabetic and antihyperlipidemic activities. Bourjot et al., [37] has isolated prenylated flavonoids like styracifolins, heterophyllin, artonins and artoheterophyllin with strong antiplasmodial and antitrypanosomal activities from the stem bark of *Artocarpus styracifolius.* Recently, Maneechai et al., [38] have reported the presence of certain prenylated derivatives of 5,7,2′,4′-tetrahydroxyflavones and 2,4,3′,5′-tetrahydroxystilbene (oxyresveratrol) with potent free radical scavenging activities from the Callus cultures of *Artocarpus lakoocha.* Compounds like artocarpusins A, B and C, chalcone and flavone, respectively, and known flavonoids with significant anticancer activities were isolated from the twigs of *Artocarpus heterophyllus*[39].

**Figure 4:**
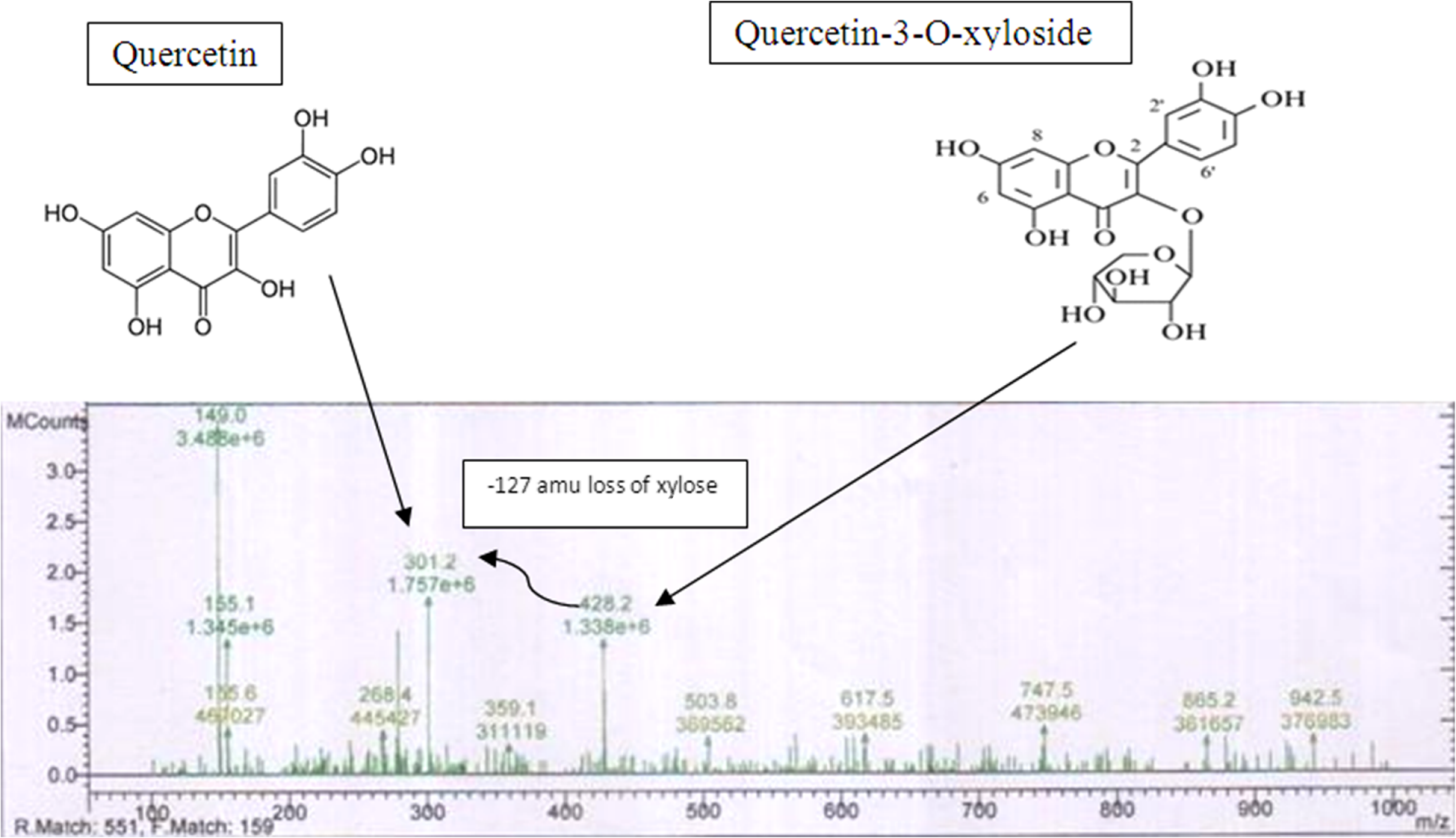
**Full scan ESI/MS spectra of parent ion of at *m/z *** **428.2 and fragment ion at *m/z *** **301.2 (quercetin).**

Previous scientific documentation by [40-44] on the identification and fragmentation pattern of flavonoids analyzed through LC-MS have clearly supported with our present recorded results (Figure 4).

## Conclusion

From the present study, it was found that PHWE of *Artocarpus heterophyllus* possess a strong antiglycation activity. Flavonoid compounds like rutin, Quercetin, Quercetin-3-O-xyloside etc., may be responsible for the antiglycation activity. An improved antiglycation activity in *Citrus sinensis* seeds was observed and recorded after the CCD based RSM optimization process. Finally, it was concluded that both *Artocarpus heterophyllus* and *Citrus sinensis* can be used as a classical herbal medicine in the management of chronic diabetes mellitus.

## Competing interests

The authors declare that they have no competing interests.

## Authors’ contributions

AS - Postgraduate student carried out the project work. TS - Project supervisor, framed the work design, contributed RSM design and LC-MS analysis, and prepared the manuscript. KK - First project review committee member and contributed extraction process. VS -Second project review committee member and contributed extraction process. All authors read and approved the final manuscript.
